# Editorial: Understanding of brain cellular dysfunction in psychiatric disorders-relevant phenotypes: from humans to models

**DOI:** 10.3389/fpsyt.2024.1487010

**Published:** 2024-09-11

**Authors:** Giulia Poggi, Anes Ju, Esther Castillo-Gómez

**Affiliations:** ^1^ Department of Psychiatry and Psychotherapy & Institute of Anatomy, University Medical Center of the Johannes Gutenberg-University Mainz, Mainz, Germany; ^2^ Samsung Advanced Institute of Technology (SAIT), Samsung Electronics, Suwon-si, Republic of Korea; ^3^ Department of Medicine, School of Medical Sciences, Universitat Jaume I, Castelló de la Plana, Spain; ^4^ Spanish National Network for Research in Mental Health (CIBERSAM), Instituto de Salud Carlos III, Madrid, Spain

**Keywords:** mental disorders, excitatory neurons, neurotrophic factors, inflammasome, inhibitory neurons, glia cells

The World Health Organization (WHO) reports that one in eight individuals globally is affected by mental disorders, including psychiatric illnesses and neurodevelopmental pathologies, highlighting the profound and widespread impact of these conditions. These individuals often endure substantial cognitive, emotional, and behavioural dysfunctions, particularly when access to effective treatments is limited. The category of mental disorders encompasses a broad spectrum of pathological conditions, including schizophrenia (SCZ), depression, anxiety disorders, bipolar disorders, post-traumatic stress disorders (PTSD), eating disorders, disruptive behaviour/dissocial disorders, and neurodevelopmental disorders such as autism spectrum disorders (ASD) and attention deficit hyperactivity disorder (ADHD)^1^.

Both environmental and genetic factors, as well as their interaction, are well-established contributors to the onset of mental disorders (1). However, the inherent complexity, heterogeneity, and multifactorial nature of these disorders have hindered significant progress in pinpointing the specific molecular and cellular pathways involved. Indeed, to date, there is no consensus on the underlying pathophysiology of mental disorders.

To address this challenge and advance the field, the National Institute of Mental Health (NIMH) introduced the Research Domain Criteria (RDoC) framework (2). Unlike traditional clinical diagnostic approaches, the RDoc framework examines mental disorders by assessing the dysfunction of specific behavioural and biological systems that may be shared across different conditions.

Inspired by this innovative approach, we have assembled a collection of preclinical and clinical studies focused on cell type-specific functions that are implicated in the onset or exacerbation of psychiatric symptoms. This Research Topic includes three research articles (Wei et al.; Pan et al.; Buttermore et al.) and one review (Hassamal) that explore the role of excitatory neurons, neurotrophic factors and inflammasome in mental disorders. Specifically, through the implementation of induced-pluripotent stem cells (iPSCs) system, Buttermore et al. explored the impact of the 16p13.11 deletion - associated with increased risk for SCZ and ADS - on the morphology, activity, and synaptic properties of iPSCs-derived cortical neurons. Their findings revealed an increased number of Synapsin-1^+^/PSD95^+^synapses, altered neuron development-related transcriptome and synaptic formation, and increased neuronal branching in probands compared to non-carrier controls, irrespective of deletion size (Buttermore et al.).

Similarly, Wei et al. identified 247 genes associated with ADHD by integrating genome-wide association study (GWAS) data with brain expression quantitative trait locus (eQTL) data using summary-data-based Mendelian randomization (SMR). Their findings revealed that these ADHD risk genes are primarily enriched in brain tissues, particularly within the mesencephalon, visual cortex, and frontal lobe regions. Further cell-type-specific analysis indicated that these genes are highly expressed in excitatory neurons (Wei et al.). These results, although preliminary, suggest that the aetiology of ADHD may involve mechanisms related to excitatory neurons in these brain regions, potentially overlapping with the pathophysiology of conditions like SCZ and ADS.

Further, Pan et al. explored the pathophysiology of SCZ from the perspective of common variations - specifically single nucleotide polymorphisms (SNPs) - in genes encoding for brain-derived neurotrophic factor (*BDNF*) and matrix metalloproteinase-9 (*MMP-9*). Their study confirmed an increased likelihood of developing SCZ in patients carrying the *BDNF* rs6265 196 G>A (GG or GA genotypes), although the *MMP-9* rs3918242-1562 C>T SNP showed no association. Notably, *BDNF* GG and MMP-9 CC genotypes were associated with higher Positive and Negative Syndrome Scale (PANSS) scores as compared to other genotypes (Pan et al.).

Lastly, a review by Hassamal highlighted emerging inflammation-related mechanisms that connect chronic stress to depression onset, emphasising the critical role of neurotrophic factors, including BDNF, in immune regulation. This review also noted ongoing research into dipeptides that mimic BDNF and specifically target the tyrosine kinase B (TkB) receptor as potential treatments for depression (Hassamal).

While these studies underscore the importance of mechanisms relevant to mental disorders and call for further confirmatory research, it is important to recognize that they do not offer a comprehensive overview of the biological substrates currently under investigation in mental illness. For instance, substantial evidence has also consistently implicated inhibitory neurons and glia cells in the onset of mental disorders (for relevant reviews, see (3–8).

In summary, while the field of research in mental disorders has still a long way to go, a collective effort to explore relevant cellular and molecular mechanisms - possibly through the implementation of the RDoC framework rather than conventional diagnostic criteria - holds promise for unravelling the complexity of mental illnesses and determining their underlying pathophysiology. Understanding these mechanisms is crucial not only for advancing our knowledge but also for developing more effective therapeutic approaches, ultimately improving the treatment and management of mental health disorders.

## Footnotes

1
https://www.who.int/news-room/fact-sheets/detail/mental-disorders
